# Systemic lupus erythematosus presenting with status epilepticus and acute cardiomyopathy with acute heart failure: case report

**DOI:** 10.1186/s41983-020-0149-9

**Published:** 2020-01-29

**Authors:** H. M. M. T. B. Herath, Aruna Kulatunga

**Affiliations:** National Hospital of Sri Lanka, Colombo, Sri Lanka

**Keywords:** Status epilepticus, Acute cardiomyopathy with heart failure, Systemic lupus erythematosus

## Abstract

**Introduction:**

Systemic lupus erythematosus is a connective tissue disorder, which causes complex multi organ involvement. Neurological and cardiac manifestations have been well noted but complications such as status epilepticus and acute myocarditis with heart failure at presentation remains uncommon.

**Case description:**

A 15-year-old, previously healthy, South Asian, Sri Lankan female presented with status epilepticus and the seizures only responded to intravenous midazolam and thiopentone sodium. On the fourth day, she developed tachycardia and shortness of breath and was found to have cardiomyopathy with heart failure with an ejection fraction 40%. Along with a positive urinary sediment, a positive ANA with a very high level of ds-DNA and low C3 and C4 levels confirmed our suspicion of systemic lupus erythematosus.

**Discussion and evaluation:**

Systemic lupus erythematosus presents in a variety of clinical presentations and the spectrum may range from unique to ubiquitous. Clinicians should have a high index of suspicion specially when encountering atypical presentations with multi-organ involvement, especially when patients tend to be young females. Status epilepticus and myocarditis are uncommon manifestations of systemic lupus erythematosus, and should be appreciated early, as if inappropriately managed would have a deleterious impact on mortality and morbidity.

## Introduction

Systemic lupus erythematosus (SLE) is a complex connective tissue disorder, which has the propensity to cause multi organ involvement. Neurological manifestations in SLE are numerous and varied, of which central nervous system (CNS) manifestations tend to be diverse and often have major prognostic consequences. Seizures in SLE are well noted, and develop in approximately 10 to 20% of patients [1–3], either as the preliminary manifestation of the disease or occurring later.

The cardiac spectrum of presentation in SLE is broad as almost every anatomic component of the heart is involved. That being said, myocarditis happens to be an uncommon manifestation in SLE, with a noted prevalence being as low as 3%, though contradictory reports suggest that to be as high as 25% [4–7]. Myocarditis complicated with acute heart failure as an early manifestation of SLE is rarely reported in the literature.

In this case vignette, we present a young female who presented with both status epilepticus and subsequently acute myocarditis complicated with heart failure as an early and atypical presentation of SLE. We discuss similar cases reported in literature and emphasize the importance of screening for autoimmune etiology in young females who present with seizures or myocarditis without other risk factors as most of such cases were diagnosed as SLE after the acute episode.

## Case description

A 15-year-old previously healthy South Asian, Sri Lankan female presented with three episodes of generalized tonic clonic seizures over a 24-h period, which progressed to status epilepticus within 2 h of admission. A targeted history from observer and family member revealed that she had developed an erythematous painless rash over both legs that had progressed over the preceding 2 weeks prior to the presentation. Furthermore, she also had a headache for 2 days, which had been diffuse in nature without features favoring meningism, discernible systemic symptoms, or fever to suggest an association or etiology. There was no background history of autoimmune diseases. The patient and family failed to recognize the clinical significance and did not seek medical intervention when these symptoms had occurred.

On examination, she was afebrile. She had mild periorbital edema and a resolving purpuric rash was noted on both her ankles. Despite a detailed neurological examination, no focal neurological signs were demonstrable. The fundi were also normal with no papilledema. Examination of her cardiovascular system revealed her blood pressure to be within reference range; however, a tachycardia was noted, though it was regular in rhythm. The jugular venous pressure was however elevated. Auscultation revealed a grade 2 pan-systolic murmur at the apex, favoring a mitral regurgitation, but the apex was not shifted. Respiratory system examination revealed tachypnea and presence of bibasal lung crepitations, in the absence of other features. Abdominal examination failed to reveal clinically significant findings.

In view of her seizures and status epilepticus, she was treated with intravenous diazepam 10 mg followed by intravenous phenytoin 18 mg/kg bolus along with oral sodium valproate via a nasogastric tube, which failed to control seizures. She was therefore electively intubated and ventilated while inducing paralysis and was started on intravenous midazolam followed by thiopentone sodium.

Capillary blood sugar values and serum electrolytes were within normal reference range. All basic investigations including blood counts, biochemistry, and cultures were done along with a lumbar puncture. Erring on the side of caution, she was empirically treated with intravenous ceftriaxone and intravenous acyclovir along with intravenous dexamethasone to cover the possible spectrum of meningoencephalitis.

While on treatment for possible meningoencephalitis, her clinical picture deteriorated; her tachycardia worsened and desaturated on atmospheric oxygen. Worsening signs of heart failure alerted us to the possibility of myocarditis. Electrocardiogram showed sinus tachycardia with T wave inversions in chest leads V2 to V6. Despite this, her cardiac enzyme assay (Troponin T and I, CK- MB) was negative. Her chest x-ray revealed relative cardiomegaly. Transthoracic 2D-echocardiohraphy (Phillips EQIP CVx, made in Germany) revealed moderate left ventricular systolic dysfunction with an ejection fraction of 40% with left ventricular global hypokinesia, along with a grade 2 severity mitral regurgitation, grade 1 severity aortic regurgitation; a clinically insignificant thin rim of pericardial effusion with no right atrial or ventricular collapse was noted. Following a cardiac consult, a clinical decision was taken to manage her medically with diuretics and fluid restriction. Upon stabilization, she was started on bisoprolol 2.5 mg bd and ivabradine 2.5 mg daily for control of heart rate along with a low dose enalapril of 2.5 mg daily.

Upon clinical recovery from status epilepticus and cardiomyopathy, while still under investigation, we noted a trend toward reduction in cell counts in her whole blood analysis. Pancytopenia was seen on day 14 of her presentation. Hemoglobin dropped from 12.4 to 8.2 g/dL, while the total white blood cell count (WBC) was noted to have reduced to 3.5 × 10^9^/L (4–10) with a differential count suggestive of neutropenia (2.8 × 10^9^/L) and a lymphocyte count of 0.35 × 10^9^/L. Thrombocytopenia was also noted. Blood picture revealed few fragmented red blood cells (RBC) with the lactate dehydrogenase elevated at 851 U/L (225–450). Coomb’s test was negative. Ongoing inflammation was appreciated, as the C-reactive protein was initially elevated at 60 mg/l (< 5), which then decreased to 8 mg/l (< 5). The erythrocyte sedimentation rate (ESR) also rose from 22 mm in the first hour, to reach 50 mm later in the second week of her presentation. Her coagulation profile (prothrombin time/international normalized ratio, activated partial thromboplastin time and plasma fibrinogen, renal and thyroid function tests, and liver profile) were all normal (Table 1).

**Table 1:** Basic investigations

Investigation	Normal range	Investigation	Normal range
Serum creatinine = 0.73 mg/dL	0.68–1.36 mg/dL	Serum sodium = 138 mmol/L	135–145 mmol/L
Serum potassium = 3.9 mmol/L	3.5–5.1 mmol/L		
Serum albumin =34 g/L	36–50 g/L	Globulin = 28.0 g/L	22–40 g/L
Serum alkaline phosphatase = 37 U/L	30–120 U/L	GGT = 40 U/L (< 55)	< 55 U/L
ALT = 37 U/L	< 50 U/L	AST = 46 U/L	< 50 U/L
Total bilirubin = 14 μmol/L	5–21 μmol/L		
Serum iron = 100.9 μg/dL	59–156 μg/dL	T.I.B.C. = 349	291–430
Transferrin saturation = 30.8%	20%–50%	Ferritin = 388 ng/mL	28–365 ng/mL
TSH = 0.813 μIu/mL	0.5–4.5	Free T4 = 1.76 ng/dL	0.78–2.19 ng/dL
Serum ionized calcium 1.12: 1.09: 1.11 (mmol/L)	1.12–1.32 mmol/L	Serum magnesium 1.91: 1.77: 2. 11 mg/dL	1.58–2. 55 mg/dL
Urinary calcium/creatinine ratio 0.325 mmol/g	0.225–8.2 mmol/g	Urinary magnesium/creatinine ratio 1.03 mmol/g	
24 h urinary calcium 2.5 mmol	2.5–7.5 mmol/ day	24 h urinary magnesium 1.15 mmol/day	
Intact PTH = 70.2 pg/ml	14–72 pg/ml		

In suspecting a multisystem disease, three urinalysis reports done within the first 2 weeks repeatedly showed moderately field full RBC in a high power field with 40% dysmorphic RBC, red cell casts, and WBC (25–30 cells/per high power field). Despite this, her urine culture was sterile. She also had a sub-nephrotic proteinuria (600 mg of protein for 24 h). Ionized calcium and serum magnesium were repeatedly marginally below the reference value with normal urinary excretion of calcium and magnesium. Serum parathyroid hormone level was also normal (Table 1).Vitamin B_12_ levels were normal at 273 pmol/L (140–650).

Extensive neurological investigations were carried out. Non-contrast CT brain was suggestive of mild cerebral edema. Magnetic resonance imaging with T2 flair image showed very subtle altered signal intensity in bilateral parietal and occipital regions, suggesting acute parenchymal inflammatory changes favoring encephalitis. The cerebrospinal fluid (CSF) analysis showed isolated high levels of protein (387 mg/dL) in the absence of leucocytes and red blood cells. CSF for gram stain, culture, Ziehl-Neelsen stain, polymerase chain reaction (PCR) for mycobacterium tuberculosis, tuberculosis culture, herpes simplex virus 1 PCR, Japanese encephalitis IgM antibodies, anti-NMDA (*N*-methyl d-aspartate) and receptor antibodies were all negative. Electroencephalogram revealed only diffuse slow waves.

Cultures of blood, endotracheal secretions, and urine were sterile. Leptospirosis serology (microscopic agglutination test for leptospirosis), Rickettsia serology, viral studies for HIV (human immunodeficiency virus antigen and antibody), JE (Japanese encephalitis antibodies IgG and Ig M), EBV (Epstein-Barr virus monospot and antibody test), CMV (Cytomegalovirus IgG, IgM, and PCR), and dengue were all negative.

On suspicion of connective tissue disease, anti nuclear antibodies (ANA) were done and was found to be weakly positive (1: 40) (Method used: indirect immunofluorescence assay, Substrate used HEp 2 cells). Anti dsDNA showed positivity with very high titers of > 150 IU/mL. (Positive if > 46.1 in method used ELISA, confirmed by IFA). Human complement 3 (C3) and human complement 4 (C4) were both low: C3 = 41 mg/dL (83–177), C4 = 03 mg/dL (12–36). Anti cardiolipin antibody, anti-beta 2 glycoprotein antibody, and lupus anticoagulant were negative.

Intravenous antibiotics and antivirals were ceased with negative cultures and a working diagnosis of SLE was made. Heart failure treatment was continued till she showed clinical and echocardiographic recovery. Sodium valproate was continued at a dose of 200 mg tds while phenytoin infusion was tailed off gradually. By day 14, she had recovered back to her premorbid state with complete recovery from both her neurological and cardiac conditions. Serial ECHOs were done and by day 14 the left ventricular ejection fraction had improved back to 60% with no regional wall motion abnormalities. She was discharged on day 21, on sodium valproate and folic acid with a follow up review planned by the multidisciplinary team. A repeated urinalysis on the day 21 only showed few RBC and WBC. The urine protein creatinine ratio was marginally elevated at 0.5 (< 0.4). Because of the resolving microscopic hematuria and proteinuria, renal biopsy was not done during the current admission. A repeated ANA done on day 28 was high (1/320) and the dsDNA too was elevated at 81.7 IU/mL.

## Discussion and evaluation

The diagnosis of SLE was made using the American College of Rheumatology criteria and Systemic Lupus International Collaborating Clinics criteria. Our patient had renal involvement with proteinuria and positive urinary sediments, a neurologic disorder with seizures in the absence of an alternate cause, and hematologic disorder with pancytopenia making up the clinical criteria. The immunological criteria were fulfilled, as the ANA was positive, anti-dsDNA antibody strongly positive along with low levels of complement. The diagnosis of systemic lupus erythematosus was made with confidence, while excluding alternate possibilities and was inferred to be the etiology of her unique presentation.

The rising ESR, low complement levels, and high titres of ds DNA were suggestive of ongoing active phase of the disease [8, 9]. Typically during the neurologic episodes, the ESR and plasma viscosity are elevated while C-reactive protein levels tend to be normal. Lupus-related serum antibody tests are usually supportive with cerebral lupus [10]. Majority of seizures in SLE are reported to be generalized, although both generalized and partial seizures can occur [1, 2, 11]. Seizures tend to occur early in the course of systemic lupus erythematosus [12] and in one case series, nearly 54% of patients had seizures within the first year of diagnosis of SLE [1]. Serologically, the presence of certain autoimmune antibodies heralds the risk of seizure in SLE; these include moderate-to-high titers of IgG anti cardiolipin anti bodies [2, 3, 13, 14], autoantibodies to beta-2-glycoproteins [14, 15], moderate- to high-titer serum anti-Smith antibodies [13], and anti-50 kDa antibody [16]. Though seizures are common is SLE, status epilepticus is considered a rare phenomenon either at presentation or even along the course of the disease [17, 18]. Our patient presented with status epilepticus and based on the clinical and serological findings, the active state of her disease could be taken as the cause for her presentation in status epilepticus.

According to European League Against Rheumatism recommendations, if seizures are thought to reflect an acute inflammatory event or a concomitant lupus flare, glucocorticoids alone or in combination with immunosuppressive therapy should be given [19]. The combination of intravenous methylprednisolone and intravenous cyclophosphamide was shown to be effective in refractory seizures in the context of generalized lupus activity [20]. But in our patient, because of the delayed diagnosis of SLE, steroids was not given with the intention of treating SLE. However, she was given dexamethasone intravenously as a part of meningitis treatment.

Cardiac disease is common among patients with SLE with the involvement of valves, pericardium, myocardium, and coronary arteries. Myocarditis is a rare but potentially fatal and often asymptomatic manifestation of SLE, which has an impact on damage accrual and survival [5]. In this case, we presumed the diagnosis of lupus myocarditis as she fulfilled the criteria for SLE, with most of the viral studies being negative and with no risk factors for atherosclerosis causing ischemic heart disease. Most of such cases were diagnosed on similar basis in the literature [21–25]. Treatment of lupus myocarditis has not been assessed in controlled trials since this is an infrequent manifestation of lupus. But dramatic improvement in cardiac function has been noted with aggressive therapy with oral or intravenous glucocorticoids, immunosuppressive therapy, and intravenous immunoglobulins [26–28].

The patient showed dramatic clinical improvement and by day 14 was asymptomatic. It was a challenge to decide whether the patient needed steroids or advanced immunosuppressive therapy. Her serological markers suggested active disease but not all patients with these serologic markers have active disease and these markers do not necessarily predict disease exacerbation. Also, fluctuations in laboratory test values are poor predictors of disease exacerbations in SLE [29]. Even in the presence of serological markers favoring disease, e.g., low complement and elevated anti-DNA antibody titers, patients may not have clinical evidence of active disease [30]. However, usually clinical and laboratory correlation in SLE tend to show a homogenous relationship and most SLE patients showing clinical-serological concordance with only minority showing discordance. Therefore, it is important to monitor both clinical and serological features in patients with SLE [31]. Steiman, A.J. and colleagues suggested that treatment decisions in SLE patients must rely upon close clinical observation and therapy should be adjusted if there are signs of clinical worsening of the disease [32]. Considering risk versus that of benefit, starting steroids at very young age will cause detrimental side effects long term and since she did have any clinical features of active disease, a clinical decision was taken not to start immunosuppressive therapy in our patient. Instead, we planned to monitor her closely for clinical or serological evidence of relapse and evolution. However, in the context of possible recurrence of seizures, especially with underlying SLE, she was continued on sodium valproate, with long-term neurology review and monitoring. She was reviewed recently, 6 months after the initial episode, and was doing well.

In conclusion, systemic lupus erythematosus is a complex connective tissue disorder that has the propensity to inflict multi-organ dysfunction in varied ways and can have a variety of clinical presentations. Our patient presented with status epilepticus and acute cardiomyopathy with heart failure, but showed dramatic improvement within 2 weeks. Status epilepticus and acute myocarditis with heart failure are uncommon yet potentially fatal manifestations of SLE. This case emphasizes the importance of considering autoimmune diseases into the spectrum of differential diagnosis early when atypical clinical presentations occur specially with multi-organ involvement as prompt diagnosis may help in reducing morbidity and mortality by allowing early appropriate and effective treatment.

## Data Availability

The datasets supporting the conclusions of this article are included within the article.

## Abbreviations

CNS: Central nervous system
CSF: Cerebrospinal fluid
ESR: Erythrocyte sedimentation rate
PCR: Polymerase chain reaction
RBC: Red blood cells
SLE: Systemic lupus erythematosus
WCC: White blood cell count
